# Massive marine methane emissions from near-shore shallow coastal areas

**DOI:** 10.1038/srep27908

**Published:** 2016-06-10

**Authors:** Alberto V. Borges, Willy Champenois, Nathalie Gypens, Bruno Delille, Jérôme Harlay

**Affiliations:** 1 Université de Liège, Unité d’Océanographie Chimique, Institut de Physique (B5), B-4000, Belgium; 2 Université Libre de Bruxelles, Laboratoire d’Ecologie des Systèmes Aquatiques, CP221, Boulevard du Triomphe, B-1050, Belgium

## Abstract

Methane is the second most important greenhouse gas contributing to climate warming. The open ocean is a minor source of methane to the atmosphere. We report intense methane emissions from the near-shore southern region of the North Sea characterized by the presence of extensive areas with gassy sediments. The average flux intensities (~130 μmol m^−2^ d^−1^) are one order of magnitude higher than values characteristic of continental shelves (~30 μmol m^−2^ d^−1^) and three orders of magnitude higher than values characteristic of the open ocean (~0.4 μmol m^−2^ d^−1^). The high methane concentrations (up to 1,128 nmol L^−1^) that sustain these fluxes are related to the shallow and well-mixed water column that allows an efficient transfer of methane from the seafloor to surface waters. This differs from deeper and stratified seep areas where there is a large decrease of methane between bottom and surface by microbial oxidation or physical transport. Shallow well-mixed continental shelves represent about 33% of the total continental shelf area, so that marine coastal methane emissions are probably under-estimated. Near-shore and shallow seep areas are hot spots of methane emission, and our data also suggest that emissions could increase in response to warming of surface waters.

Methane (CH_4_) is the second most important greenhouse gas (GHG) after CO_2_, accounting for 32% of the anthropogenic global radiative forcing by well-mixed GHGs in 2011 relative to 1750^1^. Yet, there remains an important uncertainty on estimates of the sources and sinks of CH_4_^2^, and how their variations can affect the atmospheric CH_4_ growth rate and burden^3^. The atmospheric CH_4_ increase (34 TgCH_4_ yr^−1^ for 1980–1989 and 6 TgCH_4_ yr^−1^ for 2000–2009^1^) is calculated from the measured increase of the CH_4_ concentration in the atmosphere, but results from the net balance between the sum of sources and of sinks which are one to two orders magnitude larger. The open ocean is a very modest source of CH_4_ to the atmosphere (0.4–1.8 TgCH_4_ yr^−1 ^4) compared to other natural (220–350 TgCH_4_ yr^−1^) and anthropogenic (330–335 TgCH_4_ yr^−1^) CH_4_ emissions^2^. Coastal regions are more intense sources of CH_4_ to the atmosphere than open oceanic waters^5^. Continental shelves emit about 13 TgCH_4_ yr^−1 ^5 and estuaries emit between 1 and 7 TgCH_4_ yr^−1 ^5,6,7,8. The high CH_4_ concentrations in surface waters of continental shelves are due to direct CH_4_ inputs from estuaries and from sediments where methanogenesis is sustained by high organic matter sedimentation^5^,^6^,^9^. Natural gas seeps from continental shelves contribute additionally between 16 and 48 TgCH_4_ yr^−1 ^10,11. Biogenic or thermogenic CH_4_ can accumulate in large quantities in sub-surface seabed (gassy sediments) in deep and shallow areas, and can be released as bubbles (gas flares) or by pore water diffusion. However, the estimates of CH_4_ “emission” from marine seeps^10^,^11^ correspond to CH_4_ release from sediments to bottom waters and not to the actual transfer from surface waters to the atmosphere, which is probably much lower^12^. Bubbles dissolve in water leading to high dissolved CH_4_ concentrations in bottom waters (from tens of nmol L^−1^ up to several μmol L^−1^), but removal by microbial CH_4_ oxidation and lateral dispersion by physical transport leads usually to much lower CH_4_ concentrations in surface waters (5–20 nmol L^−1^) even in the shallow areas of continental slopes and shelves^13^,^14^,^15^,^16^,^17^,^18^,^19^.

In this study, we report a data-set of CH_4_ concentrations in surface waters of the Belgian coastal zone (BCZ) in spring, summer and fall 2010 and 2011 (Fig. S1). This is a coastal area with multiple possible sources of CH_4_ such as from rivers and gassy sediments. The BCZ is also a site of important organic matter sedimentation and accumulation unlike the rest of the North Sea^20^.

## Results and Discussion

**Figure 1:**
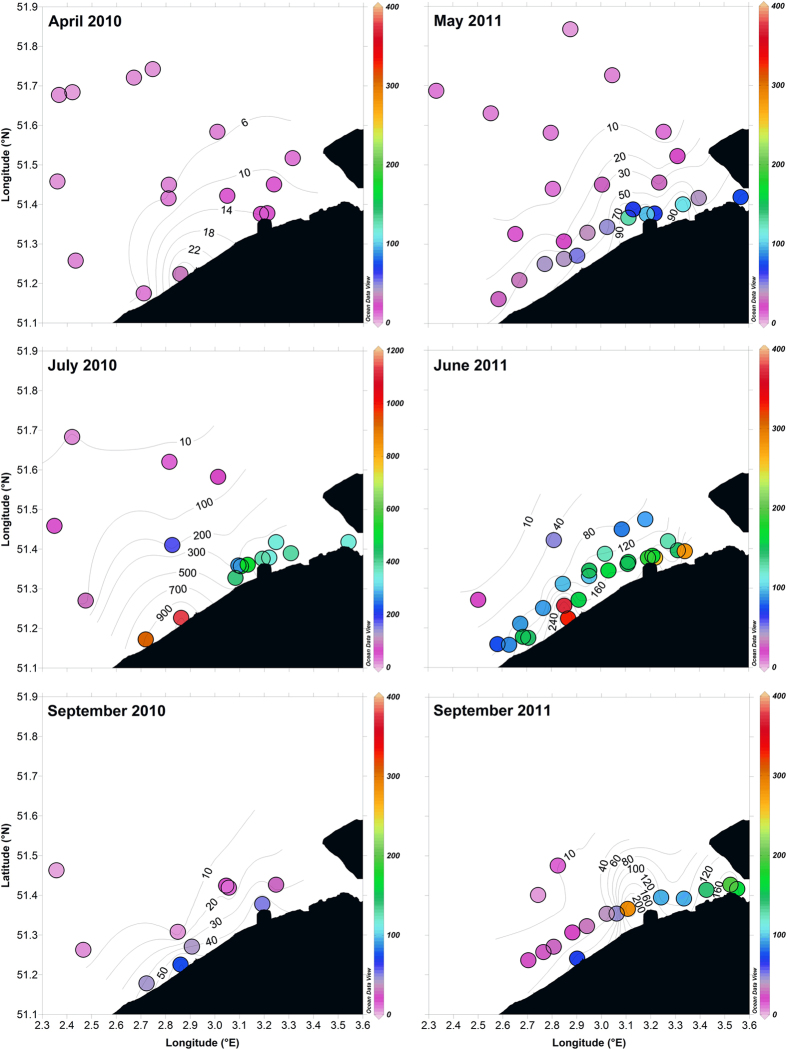
Hot-spot of dissolved CH_4_ concentration in the near-shore North Sea (up to ~300 times higher than in the open ocean). Concentration of dissolved CH_4_ (nmol L^−1^) in surface waters of the Belgian coastal zone (BCZ) in spring, summer and fall 2010 and 2011. Note the different color scale in July 2010 compared to the other cruises. Figure was produced by authors using Golden Software Surfer version 8.03 (http://www.goldensoftware.com/) and Ocean Data View version 4.6.3.1 (https://odv.awi.de/).

The CH_4_ concentrations in surface waters of the BCZ in spring, summer and fall 2010 and 2011 (Fig. 1) were high, with about 43% of the observed values above 50 nmol L^−1^, and a maximum concentration of 1,128 nmol L^−1^ in July 2011. The near-shore area (within 15 km of the coastline) was characterized by CH_4_ concentrations in surface waters between 3 and 13 times higher than the more off-shore area (>15 km away from the coastline). The overall average CH_4_ concentration in the BCZ near-shore area (139 nmol L^−1^) was ~6 times higher than in the off-shore area (24 nmol L^−1^), and in both areas distinctly above atmospheric equilibrium (~2 nmol L^−1^). These values are one to two orders of magnitude higher than the CH_4_ concentrations in surface waters of most of the North Sea with values typically <5 nmol L^−1 ^5,9 that are mainly influenced by inputs of water from the North Atlantic, where CH_4_ is close to atmospheric equilibrium^4^. Values in the BCZ were also high compared to estuarine plumes of the North Sea where maximal CH_4_ concentrations in surface waters range between 60 and 90 nmol L^−1^, such as for the Elbe^9^ and the Rhine^6^,^21^. Our own CH_4_ data in the Thames river plume were below 25 nmol L^−1^ (Fig. S2), distinctly lower than values in the BCZ. Values in BCZ were consistent with the high values (up to 372 nmol L^−1^) reported^22^ further north along the Dutch coast in March 1989 in a near-shore area with similar settings (well mixed waters overlying peat-rich sediments). The highest CH_4_ concentration in the BCZ (1,128 nmol L^−1^) was higher than any other previous report in (natural) surface waters of the North Sea, and nearly equals the value reported above an abandoned borehole in the Northern North Sea of 1,453 nmol L^−1 ^9. The highest CH_4_ concentration in the BCZ is comparable to the maximal value in surface waters (~1,800 nmol L^−1^) in the Santa Barbara Channel (Coal Oil Point), one of the most intense marine seep area in the world^13^.

**Figure 2:**
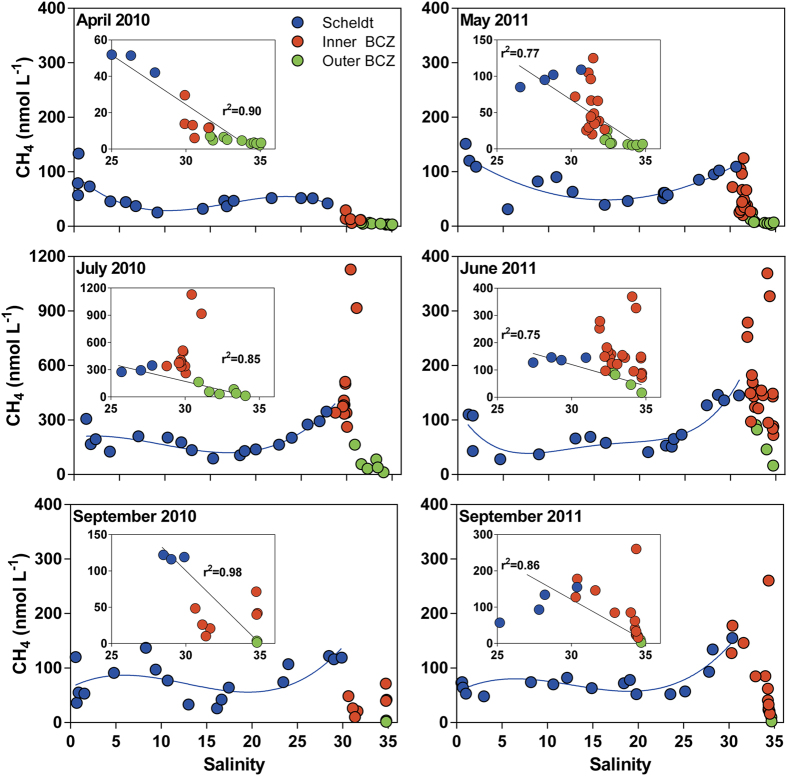
Estuarine inputs do not explain the high CH_4_ concentrations in the near-shore North Sea. Concentration of dissolved CH_4_ in surface waters of the Scheldt estuary, the near-shore Belgian coastal zone (BCZ) (<15 km from coastline) and off-shore BCZ (>15 km from coastline) in spring, summer and fall 2010 and 2011. The insert shows data at salinity >25 and the linear regression between the lower Scheldt and the off-shore BCZ data. Note the different Y-axis scale in July 2010 compared to the other cruises.

High CH_4_ concentrations in near-shore coastal areas have been frequently attributed to estuarine inputs of CH_4_^6^,^9^,^21^,^22^. This could explain the higher CH_4_ concentrations in the lower salinity region of the Thames river plume (Fig. S2). The inputs from Scheldt estuary have been shown to influence a variety of biogeochemical variables in the BCZ, such as CO_2_ 23. However, during most cruises, maximal CH_4_ concentrations measured in the BCZ were not located at the mouth of the Scheldt estuary (Fig. 1), and were higher than in the freshwater region of the Scheldt estuary (Fig. 2). Also, the CH_4_ concentrations in the near-shore BCZ were above the theoretical dilution line between the lower Scheldt (salinity >25) and the outer BCZ (Fig. 2), except for April and September 2010. This indicates that a local additional source of CH_4_ contributes to the observed high values in the near-shore BCZ.

Extensive areas of the North Sea have sediments with seismic/acoustic characteristics indicative of shallow gas accumulation, that is assumed to be mainly CH_4_ 24. In the BCZ, a four to twelve km wide band parallel to the coastline contains sediments with shallow gas, associated to a peat-rich layer from the late Pleistocene^25^. The high near-shore CH_4_ concentrations in surface waters were observed within this band of gassy sediments (Figs 1 and S1) that was most probably the source of CH_4_. The nearshore BCZ has similar sediment characteristics than Norton Sound (Alaska), an area of intense shallow submarine gas seepage^26^. However, occurrence of actual gas flaring has not been investigated in the BCZ in a systematic way, but there are some indications of local seepage of bubbles^25^. In the Scheldt estuary, an increase of CH_4_ was observed in the lower estuary (salinity >25) compared to the mid estuary (salinity ~15) (Fig. 2) which has been attributed to the presence of extensive tidal flats^7^, where gassy sediments also occur^27^. Hence, CH_4_ seepage from shallow gassy sediments could be the main reason for elevated CH_4_ concentrations in surface waters of both the nearshore BCZ and lower Scheldt.

Concentrations of CH_4_ between 15 to 300 nmol L^−1^ have been reported in bottom waters at Tommeliten, a prominent CH_4_ macro-seep area in the Central North Sea^17^, yet, in surface waters, CH_4_ concentrations were below 5 nmol L^−1^. This was attributed to removal by microbial CH_4_ oxidation and lateral dispersion by physical transport, favored by thermal stratification^17^. Similarly, in another gas seepage area in the North Sea, south of the Dogger Bank, surface waters were characterized by lower concentrations (4–518 nmol L^−1^) than bottom waters (40–1,628 nmol L^−1^)^14^.

**Figure 3:**
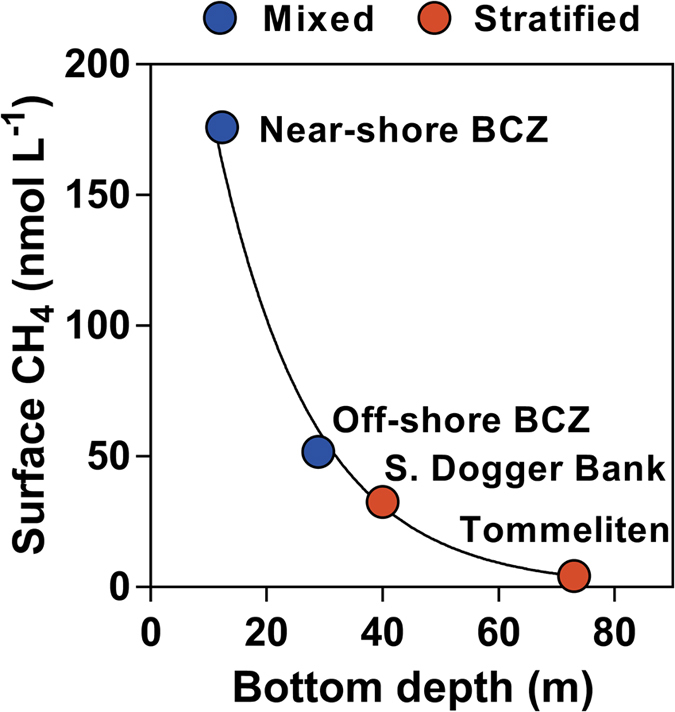
Depth controls stratification and dissolved CH_4_ levels across the North Sea. Median CH_4_ in surface waters in summer at the near-shore and off-shore Belgian coastal zone (BCZ) (<15 km and >15 km from coastline, respectively), south of the Dogger Bank^14^ and Tommeliten^17^ as a function of bottom depth. The water column is vertically homogeneous (mixed) in the BCZ and seasonally thermally stratified in the other two North Sea sites. Solid line corresponds to fit CH_4_ = 341*exp(−0.06*depth) (r^2^ = 0.996).

Due to the shallowness (<30 m) and strong tidal currents, thermal or haline stratification never occurs in the BCZ (Fig. S3). Due to the strong tidal currents, dissolved O_2_ values remain close to atmospheric equilibrium (Fig. S4), with no gradients between surface and bottom. The O_2_ and CH_4_ concentrations were uncorrelated. While CH_4_ in bottom waters was statistically higher than in surface waters (Wilcoxon matched-pairs signed rank test p = 0.0002, n = 48), the difference was very small (on average ~14%) (Fig. S3). Hence, due to the shallowness and well-mixed water column there is little loss of CH_4_ between bottom and surface waters unlike deeper and stratified areas such as Tommeliten and south of the Dogger Bank. Indeed, summertime average CH_4_ concentration in surface waters showed a regular decreasing pattern across the North Sea as a function of depth, from the vertically mixed BCZ towards the stratified and deeper regions south of the Dogger Bank and Tommeliten (Fig. 3).

**Figure 4:**
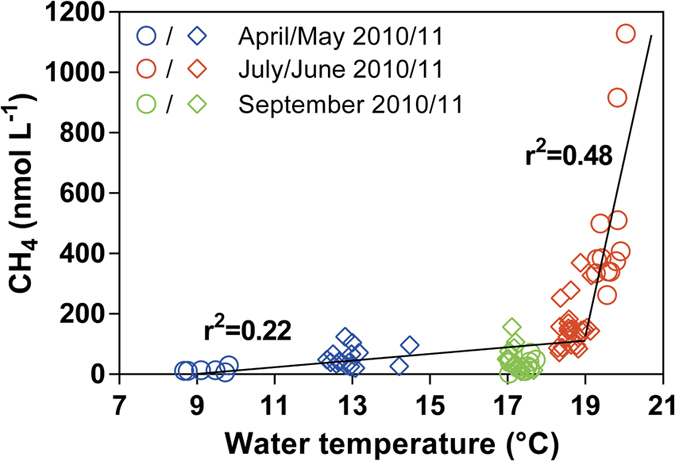
Increasing temperature enhances dissolved CH_4_ levels in the near-shore North Sea. Concentration of dissolved CH_4_ in surface waters of the near-shore Belgian coastal zone (BCZ) as a function of temperature in spring, summer and fall 2010 and 2011. Solid lines indicate the linear regressions for data < and >19 °C.

The dissolved CH_4_ concentration in the BCZ showed distinct seasonal variations with higher values in summer than spring and fall. Inter-annual variations were also observed with higher values in summer 2010 than 2011, but conversely lower values in spring and fall 2010 than 2011 in the near-shore area (Fig. 1; Table 1). In the near-shore BCZ, the lower CH_4_ concentrations were associated with lower water temperatures (April 2010) and the highest CH_4_ concentrations were associated with the higher water temperatures (June 2010) (Fig. 4). The relationship between CH_4_ concentration and temperature was non-linear with distinctly different slopes of the linear regressions for data above and below 19 °C. We interpret the positive relationship between dissolved CH_4_ and water temperature as resulting from enhanced CH_4_ release from the seafloor in response to warming. Due to the well-mixed nature of the water column in the BCZ, the amplitude of the seasonal variation of temperature in bottom waters was very large (~15 °C)^23^ compared to bottom waters in seasonally thermally stratified regions (~1 °C). In Cape Lookout Bight, enhanced bubble accumulation in sediments as well as CH_4_ diffusion and ebullition were observed in summer^28^. Increase in temperature stimulates microbial CH_4_ production^29^ and decreases CH_4_ solubility^30^, both processes contributing to releasing CH_4_ from sediments to the water column. Hence, increasing temperature could enhance a passive release of CH_4_ from gassy sediments due to the decrease of gas solubility, but this does not exclude an increase of CH_4_ production by methanogens also in response to higher temperature, and organic matter availability. Indeed, the maximal CH_4_ concentrations were observed in summer, when the sediment was enriched in organic matter produced by spring phytoplankton bloom.

The air-sea CH_4_ emissions ranged seasonally between 1 and 160 μmol m^−2^ d^−1^ in the off-shore BCZ and between 2 and 426 μmol m^−2^ d^−1^ in the near-shore BCZ (Table 1). Wind speed was lower during summer than during the other two seasons, yet, seasonal variations of the air-sea CH_4_ emissions were mainly driven by variations in CH_4_ concentrations rather than wind speed (Fig. S5). Annual air-sea CH_4_ emissions in the off-shore BCZ were 14 and 30 μmol m^−2^ d^−1^ in 2010 and 2011, respectively. These values are similar to the range of global average flux values in continental shelves of 22 to 37 μmol m^−2^ d^−1^ 5. However, the annual air-sea CH_4_ emissions in the near-shore BCZ of 126–134 μmol m^−2^ d^−1^ are ~4 times higher than the global average of continental shelves (22–37 μmol m^−2^ d^−1^)^5^ and ~370 times higher than the global average of open oceanic waters (0.2–0.5 μmol m^−2^ d^−1^) 4. Annual air-sea CH_4_ emissions in the near-shore BCZ nearly equal the CH_4_ emission of 180 μmol m^−2^ d^−1^ in Santa Barbara Channel (Coal Oil Point), one of the most intense marine seep area in the world^13^.

To envisage the impact of our findings on the marine CH_4_ emission budget, it is necessary to evaluate the representativeness of our study site for coastal areas in general. This is not an easy task since there are no global spatial datasets of gassy sediments and of submerged peat deposits. Regions corresponding to drowned coastlines (drowned forests and peatland) have been identified among the coastal environments most likely to have gas-rich sediments, in addition to estuaries, bays, rias and deltas^31^. Due to the global sea-level rise of the past 20,000 yr, it is probable that most near-shore coastal areas are drowned former land and that most of the Quaternary peat layers are now inundated and situated on the continental shelf, buried under marine sediments^32^. Yet, extensive or global spatial data-sets of submerged peat deposits are unavailable because it is difficult to identify them from seismic data alone and verification is required with coring^32^. In continental shelves where the presence of gassy sediments and seepage sites have been systematically investigated, such as around the United Kingdom, very extensive areas of gassy sediments associated with Quaternary peat deposits have been mapped^33^. In addition, permanently well-mixed water columns could represent a large fraction of continental selves. By analogy with the European continental shelf, if we assume that regions shallower than 35 m are permanently well-mixed by tidal action^34^, they would represent 33% of the total surface area of continental shelves (<200 m, that is 26,400 km^2^)^35^. The distinctly different CH_4_ concentrations in well-mixed and seasonally stratified continental shelves (Fig. 3) should then be accounted when budgeting CH_4_ emissions.

These emission estimates for the near-shore BCZ are most likely underestimated since they only account for diffusive CH_4_ fluxes, although there are some indications of local seepage of bubbles^25^. While in deeper continental shelf areas CH_4_ bubbles dissolve as they rise, and dissolved CH_4_ is removed by microbial oxidation and by horizontal physical transport^17^, in very shallow areas such as the BCZ (<30 m) bubbles from seepages could avoid dissolution^36^ and be directly emitted to the atmosphere. While the emissions from seeps should be considered as natural sources in the global CH_4_ budget, our data (Fig. 4) suggest that further warming of surface waters could increase CH_4_ emissions and provide a positive feedback on warming climate. This feedback will be expected to be acute in shallow gassy areas such as the BCZ since they are natural hotspots of CH_4_ emission, and the well-mixed water column will allow an efficient propagation of additional heat to the sediment that will be buffered by seasonal thermal stratification in deeper seep areas. The increase of temperature will stimulate the biogenic CH_4_ production, as well as, decrease Henry’s constant promoting bubbling from sediments.

## Methods

Data were collected during 6 cruises in the BCZ on the *RV Belgica* during spring, summer and fall in 2010 and 2011 (BE2010/11 – 19-23/04/2010, BE2010/18 – 05-08/07/2010, BE2010/23 – 13-16/09/2010, BE2011/13 – 02-05/05/2011, BE2011/19 – 04-07/07/2011, BE2011/24 – 12-15/09/2011) (Fig. S1). Near simultaneous data were also collected in the Scheldt estuary on the *RV Luctor* (06-07/04/2010, 12-13/07/2010, 20-21/09/2010, 09-10/05/2011, 20/06-21/06/2011, 12-13/09/2011) (Fig. S1). Sampling was carried out with a 10L Niskin bottle coupled to a conductivity-temperature-depth (CTD) probe (Sea-bird SBE19 on the *Belgica* and YSI 6600 on the *Luctor*), in surface waters (1 m depth) and on some occasions ~3 m above the seafloor. When CTD data were unavailable on the *Belgica*, we used salinity and temperature measurements from an underway instrument (Sea-bird SBE21) connected to a seawater supply (pumped at 2.5 m). Water samples were collected in borosilicate serum bottles (50 ml) with a tubing, left to overflow, poisoned with a saturated solution of HgCl_2_ (100 μl), sealed with a butyl stopper, crimped with an aluminum cap, and stored at ambient temperature in the dark until analysis. Dissolved oxygen was measured by titration with the Winkler method^37^.

The concentration of CH_4_ was determined with the headspace equilibration technique (20 ml N_2_ headspace in 50 ml serum bottles and overnight equilibration in a thermostated bath after initial manual vigorous shaking) and a gas chromatograph^38^ equipped with a flame ionization detector (SRI 8610C) calibrated with CH_4_:CO_2_:N_2_O:N_2_ mixtures (Air Liquide Belgium) of 1, 10 and 30 ppm CH_4_. Each of the three standards was analyzed in triplicate at the start and the end of the daily batch of samples (typically 30) and the calibration curve was computed by linear regression forced through zero (r^2^ ≥ 0.999). The slope of the calibration regression line was interpolated linearly from initial and final values for the whole batch of samples, although no statistical difference was ever observed between the start and end calibrations. About 10 ml of the headspace (or standard) was injected through a 6-way valve from which a 2 ml subsample (loop) was injected into a 2 ml column of magnesium perchlorate (water vapor trap), and then into a packed column (Hayesep D, 5.0 m length, mesh 80/100) kept at 50 °C, using N_2_ as carrier gas. The 10 ml volume of headspace was sampled with a plastic syringe with a steel needle through the septum, and the retrieved gas volume was replaced by a hyper-saline solution (about 60 g NaCl L^−1^) injected with another syringe in the bottom of the serum bottle, in order to keep the sampled gas sample at atmospheric pressure. Chromatographic peak areas were integrated and logged using the Peaksimple software (version 4.44 for Windows^TM^ XP). The *in-situ* CH_4_ concentration was computed^39^ from the volume of water and headspace (determined from the weight of bottles empty, and before and after making the headspace), the measured partial pressure of CH_4_ and Henry’s constant^40^. Precision estimated from multiple injections of gas standards was better than ±3.0% for the 1 ppm standard and better than ±0.5% for the other two standards. The precision estimated from duplicated samples was ±3.9%.

The air-sea CH_4_ flux (*F*) was computed according to:


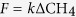


where *k* is the gas transfer velocity and ΔCH_4_ is the air-sea CH_4_ concentration gradient computed from the measured dissolved CH_4_ concentration in seawater and the concentration at equilibrium with an atmospheric CH_4_ partial pressure value of 1.8 ppm, computed with Henry’s constant^40^.

The *k* values were computed from the parameterization as a function of wind speed based on dual deliberate tracer (^3^He/SF_6_) experiments in the Southern Bight of the North Sea^41^, and the Schmidt number of CH_4_ in seawater computed from temperature^42^. Wind speed data were obtained from the National Centers for Environmental Prediction reanalysis daily averages surface flux (http://www.cdc.noaa.gov/) at 2 grid points covering the sampled region (3.7500°E 52.3799°N; 0.0000°E 50.4752°N). *F* was computed using daily wind speed values (average of the 2 grid points) for a time interval of 30 days centered on the date of the middle of the cruises.

## Additional Information

**How to cite this article**: Borges, A. V. *et al*. Massive marine methane emissions from near-shore shallow coastal areas. *Sci. Rep.* **6**, 27908; doi: 10.1038/srep27908 (2016).

## Supplementary Material

Supplementary Information

Supplementary Dataset 1

## Figures and Tables

**Table 1:** Wind speed and air-sea CH_4_ fluxes in the near-shore (<15 km from coastline) and off-shore (>15 km from coastline) Belgian coastal zone (BCZ) in spring, summer and fall 2010 and 2011 (mean ± standard deviation).

		Wind speed	Near-shore air-sea CH_4_ flux	Off-shore air-sea CH_4_ flux
		(m s^−1^)	(μmol m^−2^ d^−1^)	(μmol m^−2^ d^−1^)
2010	Spring	4.8 ± 2.3	13.9 ± 9.6	2.1 ± 1.8
	Summer	3.3 ± 2.2	426.0 ± 230.8	52.0 ± 46.7
	Fall	6.1 ± 2.1	65.7 ± 50.1	0.9 ± 3.5
	Annual	–	126.4 ± 236.4	13.7 ± 46.8
2011	Spring	5.4 ± 2.3	83.3 ± 49.6	10.6 ± 10.3
	Summer	5.2 ± 2.5	283.3 ± 141.4	100.1 ± 61.2
	Fall	5.8 ± 3.0	169.6 ± 158.4	8.5 ± 11.5
	Annual	–	134.1 ± 218.0	29.8 ± 63.1

Annual fluxes were calculated assuming a zero flux in winter (based on the very low CH_4_ concentrations measured at low temperature, Fig. 4).
