# Supplementary material for: Massive marine methane emissions from near-shore shallow coastal areas
Source: Sci Rep. 2016 Jun 10;6:27908. doi: 10.1038/srep27908 (PMC4901272; doi:10.1038/srep27908)
Supplement: Supplementary Information [file srep27908-s1.doc]

**Massive marine methane emissions from near-shore shallow coastal areas**

Alberto V. Borges^1,*^, Willy Champenois^1^, Nathalie Gypens^2^, Bruno Delille^1^, Jérôme Harlay^1^

^1^ Université de Liège, Unité d’Océanographie Chimique, Institut de Physique (B5), B-4000, Belgium

^2^ Université Libre de Bruxelles, Laboratoire d’Ecologie des Systèmes Aquatiques, CP221, Boulevard du Triomphe, B-1050, Belgium

*e-mail: alberto.borges@ulg.ac.be

**Figure S1:** Map of the study zone at the scale of Europe and the North Sea (a), and of sampling stations in the BCZ (b) and Scheldt estuary (c) in spring, summer and fall 2010 and 2011. The grey area corresponds to acoustical turbid (gassy) sediments mapped by Missiaen *et al.*^25^ in the area [2.92-3.42°E;51.25-51.50°N], smaller than our study zone, meaning that the band of gassy sediments probably extends further west along the coastline. The stations in the Scheldt estuary shown in plot c were all systematically sampled in spring, summer and fall 2010 and 2011. T = Tommeliten, DB = Dogger Bank. Figure was produced by authors using Golden Software Surfer version 8.03 (<http://www.goldensoftware.com/>).

**Figure S2:** Map of sampling stations and concentration of dissolved CH_4_ in surface waters as function of salinity in the Thames estuarine plume in April and July 2010, and May and September (Sept.) 2011. The rectangle indicates the study zone in the Belgian coastal zone (Fig. 1). The solid lines indicate the linear regressions. Map was produced by authors using Golden Software Surfer version 8.03 (<http://www.goldensoftware.com/>)

**Figure S3:** Comparison of salinity, temperature (April 2010, May 2011, June 2011) and dissolved CH_4_ concentration (July 2010 (n=1), May 2011 (n=22), June 2011(n=25)) in surface and bottom (~3 m above the seafloor) waters of the Belgian coastal zone.

**Figure S4:** %O_2_ (%) in surface waters of the Belgian coastal zone in spring, summer and fall 2010 and 2011. Figure was produced by authors using Golden Software Surfer version 8.03 (<http://www.goldensoftware.com/>) and Ocean Data View version 4.6.3.1 (<https://odv.awi.de/>).


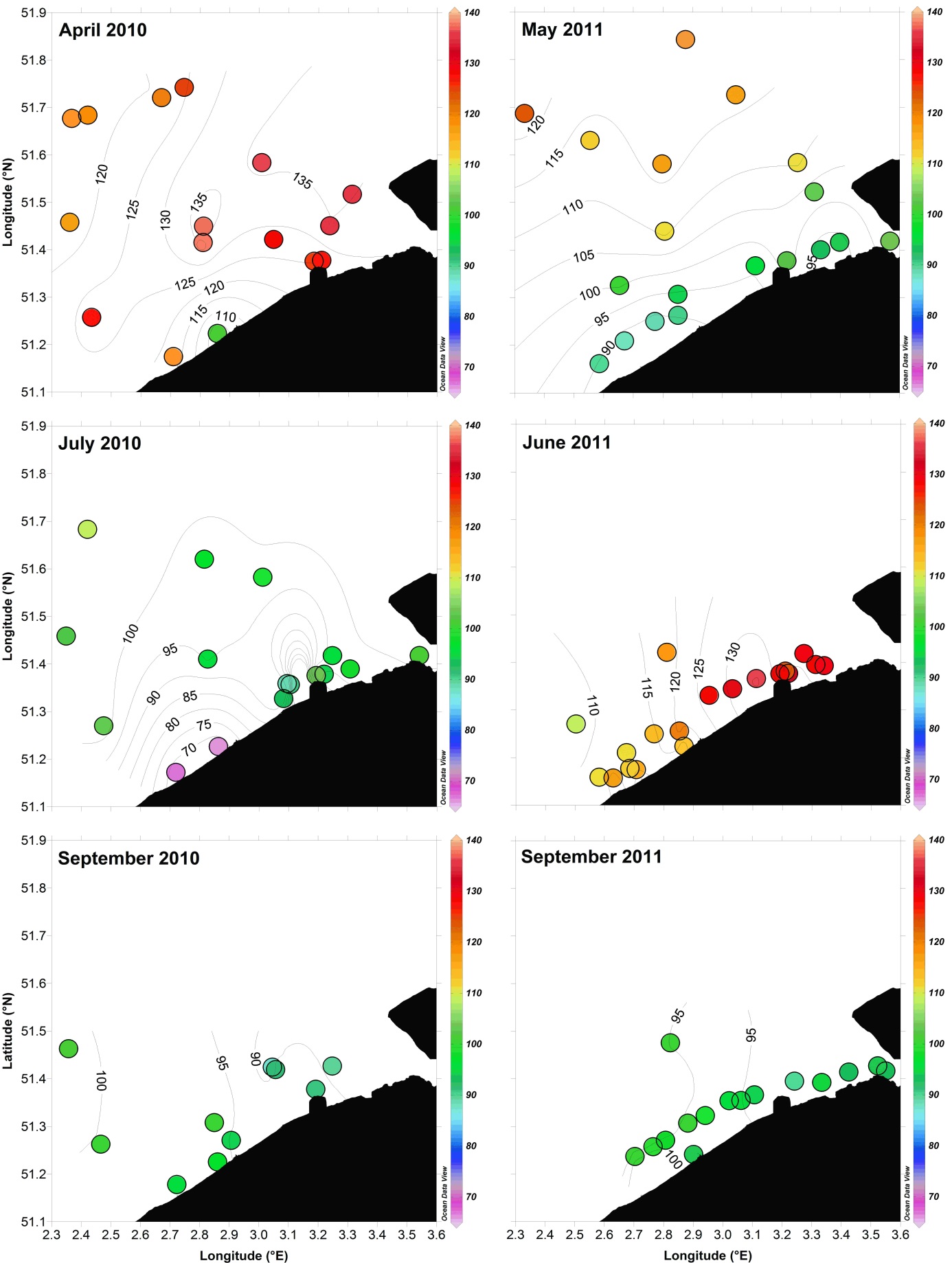


**Figure S5:** Relation between the air-sea CH_4_ flux (µmol m^-2^ d^-1^) and CH_4_ concentration (nmol L^-1^) and wind speed (m s^-1^) in the near-shore and off-shore regions of the Belgian continental shelf (BCZ) in spring, summer and fall 2010 and 2011. The lack of relationship between air-sea CH_4_ flux and wind speed, and the positive relationship between air-sea CH_4_ flux and CH_4_ concentration indicates that in the BCZ the main driver of seasonal variations of air-sea CH_4_ fluxes are the seasonal variations of CH_4_ concentration. In fact, there is a negative tendency between air-sea CH_4_ flux and wind speed, since the highest CH4 concentrations (and fluxes) are observed in summer, when wind speed is lowest.
